# Supplementary material for: Splice-Junction-Based Mapping of Alternative Isoforms in the Human Proteome
Source: Cell Rep. Author manuscript; Available in PMC 2020 Jan 15. (PMC6961840; doi:10.1016/j.celrep.2019.11.026)
Supplement: 3 [file NIHMS1546469-supplement-3.zip › DF2/PXD0010154/Liver-273-Q96QZ7-QQKPLEECEDDELPAGWEK.pdf]

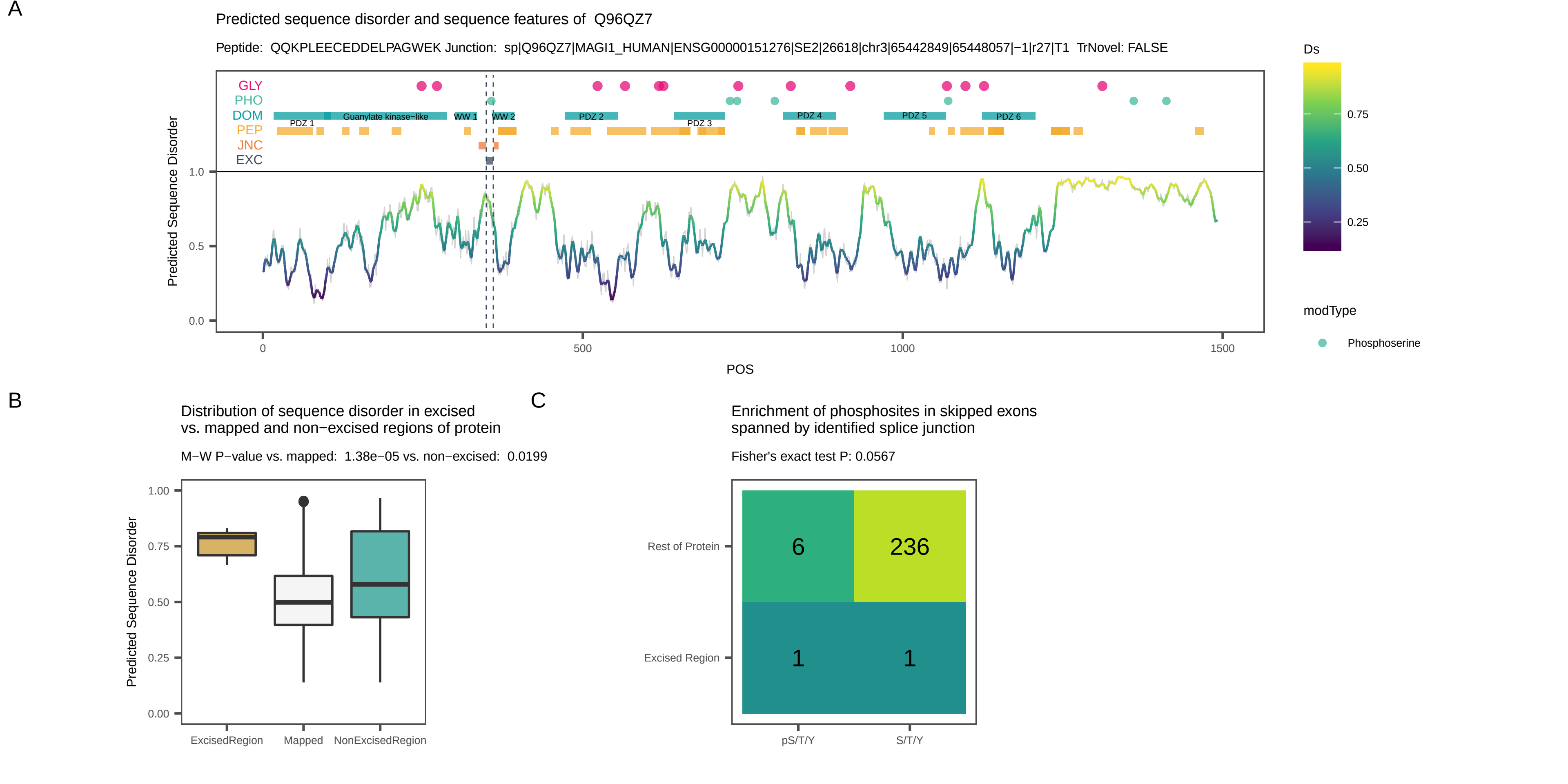
A
Predicted sequence disorder and sequence features of Q96QZ7
Peptide: QQKPLEECEDDELPAGWEK Junction: sp|Q96QZ7|MAGI1_HUMAN|ENSG00000151276|SE2|26618|chr3|65442849|65448057|−1|r27|T1 TrNovel: FALSE
GLY
PHO
DOM
PEP
JNC
EXC
PDZ 1
Guanylate kinase−like
WW 1
WW 2
PDZ 2
PDZ 3
PDZ 4
PDZ 5
PDZ 6
Predicted Sequence Disorder
1.0
0.5
0.0
0
500
1000
1500
POS
Ds
0.75
0.50
0.25
modType
Phosphoserine
B
Distribution of sequence disorder in excised
vs. mapped and non−excised regions of protein
M−W P−value vs. mapped: 1.38e−05 vs. non−excised: 0.0199
Predicted Sequence Disorder
1.00
0.75
0.50
0.25
0.00
ExcisedRegion
Mapped
NonExcisedRegion
C
Enrichment of phosphosites in skipped exons
spanned by identified splice junction
Fisher's exact test P: 0.0567
Rest of Protein
Excised Region
6
236
1
1
pS/T/Y
S/T/Y
